# The influence of childhood socioeconomic status on non-communicable disease risk factor clustering and multimorbidity among adults in Botswana: a life course perspective

**DOI:** 10.1093/inthealth/ihac026

**Published:** 2022-05-05

**Authors:** Mpho Keetile, Gobopamang Letamo, Kannan Navaneetham

**Affiliations:** Department of Population Studies, University of Botswana, Private Bag UB 00705, Gaborone, Botswana; Department of Population Studies, University of Botswana, Private Bag UB 00705, Gaborone, Botswana; Department of Population Studies, University of Botswana, Private Bag UB 00705, Gaborone, Botswana

**Keywords:** Botswana, childhood socioeconomic status, life course, multimorbidity, NCD risk factors

## Abstract

Childhood socioeconomic circumstances have a great influence on the health of individuals in adult life. We used cross-sectional data from a non-communicable disease (NCD) survey conducted in 2016, and respondents aged ≥15 y were selected from 3 cities/towns, 15 urban villages and 15 rural areas using a multistage probability-sampling technique. The total sample for the study was 1178. Two multinomial logistic regression models were fitted to data to ascertain the association between childhood socioeconomic status (SES) and NCD risk factor clustering and multimorbidity, using SPSS version 27. All comparisons were considered to be statistically significant at a 5% level. The prevalence of multiple NCD risk factors and multimorbidity was 30.1 and 5.3%, respectively. The odds of reporting NCD risk factor clustering were significantly high among individuals who reported low (adjusted OR [AOR]=1.88, 95% CI 1.21 to 2.78) and middle (AOR=1.22, 95% CI 1.02 to 2.05) childhood SES compared with high childhood SES. Conversely, individuals from a low SES background were more likely to report both single (AOR=1.17, 95% CI 1.00 to 2.01) and multiple NCD conditions (AOR=1.78, 95% CI 1.11 to 2.68) compared with those with a high childhood SES background. There is a need to stimulate policy debate and research to take cognisance of childhood socioeconomic circumstances in health policy planning.

## Introduction

Recently, most researchers have adopted the life course perspective approach to explain how socioeconomic factors at different levels of an individual's life course influence health in later years.^1^ There is abundant evidence showing a correlation between childhood socioeconomic status (SES) and adult health.^1–4^ According to these studies, childhood SES is a powerful predictor of adult health. Studies by Galobardes et al.^5^ and Pollitt et al.^6^ found that individuals who have experienced poor SES during childhood were at a greater risk of cardiovascular-related mortality irrespective of adulthood SES.

Multifaceted factors such as individual, household and community level determinants influence the health of individuals across their life course.^7^ On the other hand, the availability of health services and infrastructures influence an individual's choices, skills and behaviour related to preventive care, nutrition and hygiene among others.^8^ Empirical studies have shown that individuals from a high SES background are more likely to take advantage of modern technology and are more aware of nutritional and health-related problems,^9–13^ while, by contrast, those of poor SES are less likely to take advantage of available health resources and are unable to generate resources for improved nutrition and health, hence are more prone to non-communicable disease (NCD) risk factor clustering, ultimately leading to NCD multimorbidity in adulthood.^11–14^

Although there is mounting research evidence showing that SES exposures during childhood are powerful predictors of adult health,^11^ there is limited evidence in the context of low- and middle-income countries (LMICs). Among the available studies, the findings show inconsistent nature and direction of relationship on how childhood SES affects the health of individuals during adulthood.^1,5,6^ For instance, a study by Selvamani and Arokiasamy^15^ in India and China found that parental education as a measure of childhood SES was positively associated with cognitive functioning. Moreover, a study by Selvamani and Arokiasamy^16^ in six LMICs—India, China, Ghana, Mexico, Russia and South Africa—using WHO-SAGE data concluded that the role of childhood SES remains unclear in developing countries. According to Stringhini et al.,^17^ the inconsistent findings on childhood SES and adult health in LMICs might relate to the fewer studies conducted in these countries, including their quality and methodological issues.

Moreover, the debate on how childhood socioeconomic experiences are linked to the health of individuals during adulthood is scarce in the sub-Saharan Africa region. However, life course studies on how childhood SES influences NCD risk factor clustering and multimorbidity are beginning to take ground. As far as we know this is the first study to examine the influence of childhood SES on NCD risk factor clustering and multimorbidity among adults in Botswana. An understanding of how childhood SES influences NCD risk factor clustering and multimorbidity in adulthood will serve to guide policies and programmes on childhood socioeconomic well-being.

## Conceptual framework

The life course approach aims to examine how socioeconomic and social risk factor trajectories, acting across the life of a person, influences their health.^18^ There is a general acknowledgement of the significance of childhood SES and adult characteristics in influencing health and well-being.^19^ As a result, there is a growing focus on life course determinants of health, especially for studies of long-term changes in the epidemiology of NCDs. This is research guided by the life course approach (LCA) and seeks to understand the influence of childhood SES on adult health, particularly on NCD risk factor clustering and multimorbidity in the adult population of Botswana.

**Figure 1.**
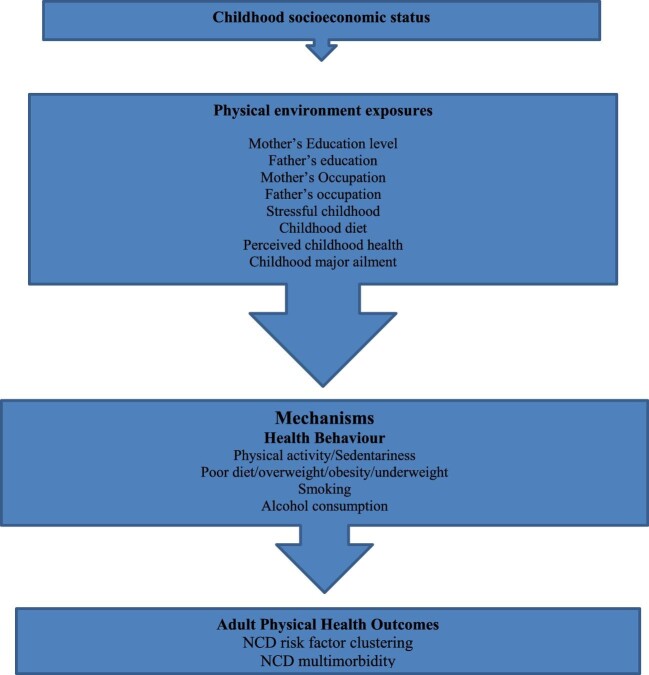
Examples of pathways that may link physical exposures associated with childhood SES to adult health (NCD risk factor clustering and NCD multimorbidity).

Figure 1 depicts the multiple interactions on how childhood SES, physical and social environments experienced during childhood have lasting effects on subsequent adult health. For example, people who experienced adverse physical environment exposures, such as coming from families with low maternal and paternal education, low-earning occupations, stressful childhood and poor childhood diet, are likely to face increased risk factors for NCDs and multimorbidity during adulthood.

This is because children of a low SES face the possibility of poor health behaviours that predispose them to NCDs during adulthood.^20,21^ Poor health behaviours predispose individuals to the possibility of poor development that ultimately leads to multiple NCD conditions.^22^ Moreover, poor health behaviours disproportionately seen among low SES adults further exacerbate poor adulthood health. For instance, a person who is exposed to NCD risk factors such as smoking, alcohol consumption, poor physical activity, poor fruit and vegetable consumption and malnutrition during childhood and adolescence faces the greatest risk of developing several NCDs later in life.^11–13^

Although we adopt this conceptual approach we are very cautious that LCA presents great challenges for the continued development of testable theoretical models and effective study design and analysis. This study is not a pure ‘life course’ study in the sense that it does not follow the same individuals throughout their entire life course, as is the case with longitudinal studies. However, although we are not tracking individuals through their life course, we track the average socioeconomic patterns for a group of individuals during childhood using self-reported health while controlling for possible confounders. As a result we are using cross-sectional data that utilised self-reports about the childhood circumstances of individuals. Previous studies have used the same approach.^23–25^

## Materials and Methods

### Study design and sample

The data used for this study were from the NCD survey conducted in March 2016. Data for the NCD study were collected using a cross-sectional design and respondents were selected through a multistage probability-sampling technique. Respondents were selected from 3 cities and towns, 15 urban villages and 15 rural areas across Botswana. Self-reported data on several NCDs and NCD risk factors as classified by the WHO ICD-10 classification of diseases and their risk factors were collected. Data were collected on social and behavioural characteristics and anthropometric measurements (such as height and weight). The WHO Study on Global Aging and Adult Health (SAGE) survey instruments were used to guide the development of the questionnaire.^26^ The total sample of the study was 1178 participants aged ≥15 y who had completed the NCD questionnaire successfully.

### Measurement of variables

#### Outcome variables

Two outcome variables were used in this study: NCD risk factor clustering and self-reported multimorbidity.

*NCD risk factor clustering:* A composite variable was created to assess the existence of clustering of NCD risk factors among study participants. The variable was created from the five NCD risk factors (tobacco use, alcohol consumption, poor physical activity, poor fruit and vegetable consumption and overweight/obesity). It was coded such that if an individual reported that there was no existence of any NCD risk factor then a code of 0 was given, if there was only one risk factor then the code was 1, or 2 if there were >1 NCD risk factors.

*Multimorbidity:* This was also a composite variable created from the list of 10 conditions—stroke, angina, diabetes, chronic lung disease, asthma, hypertension, eye/vision problems, nerves problems, skin problems and depression—classified as NCDs according to ICD-10.^11,27^ The final variable was coded such that if there was no existence of any NCD condition then a code of 0 was given, if there was any one NCD condition then the code was 1, or 2 if there were >1 NCD conditions.^11,27^ Consequently, multimorbidity is defined in this study as the presence of ≥2 chronic NCD conditions in one individual.^27^

#### Explanatory variables

A composite variable derived from the combination of material (socioeconomic) and psychosocial conditions in childhood, for example, parental education, parental occupation, perceived childhood health and childhood diet (Table 1), was used as the main explanatory variable for this study. In creating this variable we grouped positive and negative childhood socioeconomic experiences of individuals, to come up with three categories for childhood SES, namely, low=1, middle=2 and high=3. We used variables such as gender (male and female); age (≤24=1, 25–34=2, 35–44=3, 45–54=4, 55–64=5 and ≥65 y=6); education (primary or less=1 [non-formal and primary], secondary [junior and senior]=2 and tertiary and over=3); residence (cities and towns=1, urban villages=2 and rural villages=3); marital status (never married [combining never married and living together categories]=1, currently married=2 and ever married [combining divorced, widowed and separated]=3); work status (public sector [government employee]=1, private sector [non-government employee]=2, self-employed=3, not employed=4, homemaker/student=5 and retired or other [retired, non-paid family helper, house-helper, house worker]=6); and current wealth status (created from the household's ownership of durable materials using principal component analysis to derive wealth quintiles;1st to 5th quintile).

**Table 1.** Childhood socioeconomic exposure variables used to create the childhood SES index

Life-course variable	Survey question
Father's education level	The question was ‘What was the education level of your father when you were born?’ This was recoded as low education=1 (includes no education, informal education and primary) and high education level=0 (includes secondary and tertiary or high)
Mother's education level	Each respondent was asked ‘What was the education level of your mother when you were born?’ The codes were low education=1 (includes no education, informal education and primary) and high education level=0 (includes secondary and tertiary or high)
Father's occupation	State activity status and occupation of your father during your childhood. The variable was recoded as public sector=1, private sector=2, self-employed=3 and unemployed=4 (student, retired, homemaker)
Mother's occupation	State activity status and occupation of your mother during your childhood. The variable was recoded as public sector (government)=1, private sector (non-government)=2, self-employed=3 and unemployed=4 (student, retired, homemaker)
Stressful childhood	Has your life been stressful? Yes=1, no=0
Childhood diet	What kind of food was taken during childhood? Vegetarian=1, non-vegetarian=0
Perceived childhood health	How did you feel about your health? Below average=1, average=2 and above average=3
Childhood major ailment	Do you remember any major ailment you suffered? Yes=1 and no=2
Childhood SES	This was a resultant variable derived from the combination of material (socioeconomic) and psychosocial conditions in childhood (e.g. parental education, parental occupation, perceived childhood health and childhood diet). The positive and negative childhood socioeconomic experiences were grouped separately and an index was created to come up with three categories for childhood socioeconomic status: low=1, middle=2 and high=3 childhood SES

Source: Keetile et al.^13^

#### Data analysis

We first present the frequencies and percentages of the sampled participants by different background variables. We also ran bivariate analysis and χ^2^ tests between each of the independent variables and prevalence of NCD risk factor clustering and multimorbidity. A multicolliniarity test using the variance inflation factor (VIF) was undertaken to check for possible colliniarity between explanatory variables including childhood SES and current wealth status (VIF=2.207). To examine the association between childhood socioeconomic status and health, unadjusted ORs (UORs) and adjusted ORs (AORs) were derived by applying the multinomial logistic regression model. Two models were fitted to data to ascertain the association between childhood SES and health outcomes.

Model 1 is an adjusted model, in which we assessed the association between childhood SES and NCD risk factor clustering. Childhood SES is a key independent variable, and its association with NCD risk factor clustering is assessed while controlling for gender, age, education, place of residence, marital status, work status and current wealth status. Model 2 is also an adjusted model, in which childhood SES is a key independent variable, and its association with multimorbidity is assessed while controlling for gender, age, education, place of residence, marital status, work status and current wealth status.

Both models assessed the effects of childhood SES on adult health independent of (after controlling for) adult SES. The results of the logistic regression analysis are presented as AORs, together with their 95% CIs. The data were analysed using Statistical Package for Social Sciences (SPSS) version 27 (IBM Corp., Armonk, NY, USA). We used the complex samples module in SPSS to estimate the parameters of interest because the NCD survey used multistage complex deign.

## Results

### Sample description

The total sample of this study was 1178 respondents (Table 2). The majority of respondents (69.1%, 813/1178) were females, 29.5% (302/1178) were aged 25–34 y and 45.4% (534/1178) of the population resided in urban villages. Almost three-quarters (73.8%, 864/1178) of respondents were never married; over a third (35.5%, 410/1178) had primary education or less; and close to two-fifths (37.5%, 436/1178) of respondents were not employed. Wealth status was evenly distributed across the wealth quintiles, ranging from 19.9 to 20.1% (lowest, middle and highest=19.9%, while second and fourth=20.1%).

**Table 2.** Socioeconomic characteristics of the respondents

Variable	%	N
Gender		
Male	30.9	364
Female	69.1	814
Age, y		
<24	26.4	311
25–34	29.5	348
35–44	19.2	226
45–54	12.7	150
55–64	7.3	86
65+	4.9	57
Locality type		
Cities/towns	30.2	356
Urban villages	45.4	534
Rural settlements	24.5	288
Marital status		
Never married	73.8	869
Currently married	17	200
Formerly married	9.2	109
Highest level of education attained		
Primary or less	35.5	418
Junior secondary	27.2	320
Senior secondary	17.3	204
Tertiary and over	19.9	234
Work status in past 12 mo		
Public sector	10.5	124
Private sector	15.7	185
Self employed	11.2	132
Not employed	37.5	442
Homemaker-student	25.1	295
Wealth status Lowest Second Middle Fourth Highest	19.9 20.1 19.9 20.1 19.9	234 237 235 237 235
***Total***	**100.0**	**1178**

### Prevalence of NCD risk factors and multimorbidity

**Figure 2.**
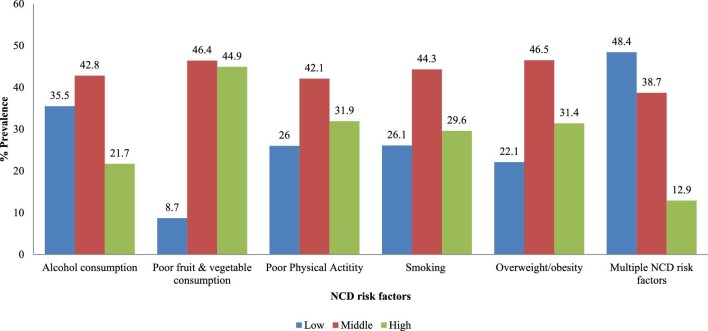
Percentage prevalence of NCD risk factors by childhood SES, Botswana, 2016.

Figure 2 presents the prevalence of NCD risk factors and their clustering by childhood SES. The prevalence of NCD risk factors was found to be high among individuals who had a middle childhood SES. For instance, alcohol consumption (42.8%), poor fruit and vegetable consumption (46.4%), poor physical activity (42.1%), smoking (44.3%) and overweight/obesity (46.5%) were significantly higher among individuals who had a middle childhood SES. However, after clustering all the NCD risk factors, it was observed that multiple NCD risk factors were significantly higher among individuals who reported low childhood SES (48.4%).

**Figure 3.**
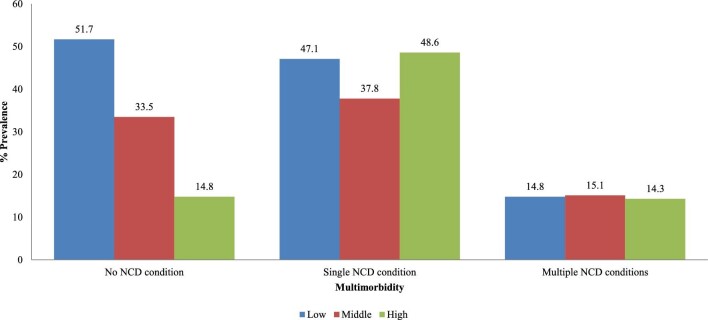
Percentage prevalence of NCD multimorbidity by childhood SES.

Figure 3 shows the prevalence of multimorbidity by childhood SES. A slightly higher proportion of people who reported middle childhood SES (48.6%) had a single NCD condition compared with those with low (47.1%) and middle (37.8%) childhood SES, while multiple NCD conditions were also slightly higher among individuals with middle childhood SES than low (14.8%) and high SES (14.3%). The overall prevalence of smoking, alcohol consumption, poor physical activity, poor fruit and vegetable consumption, overweight/obesity, multiple NCD risk factors and multiple NCD conditions was 11.5, 17.3, 48.9, 82.5, 41.4, 30.1 and 5.3%, respectively (Table 3).

**Table 3.** Prevalence of NCD risk factors and multiple risk factors

Variable	%	N
Smoking?		
Yes	11.5	136
No	88.5	1042
Alcohol consumption		
Yes	17.3	204
No	82.7	974
Poor physical activity		
Yes	48.9	576
No	51.1	602
Fruit and vegetable intake?		
Poor fruit/vegetable intake	82.5	1045
Sufficient intake	17.5	133
Overweight/obesity		
Yes	41.4	462
No	58.6	654
Multiple risk factors		
No risk factor	28.0	330
Single risk factor	41.9	494
Multiple risk factors	30.1	354
Multimorbidity		
No NCD condition	70.5	829
Single NCD condition	24.2	285
Multiple NCD conditions	5.3	63

### Association between childhood SES and NCD risk factor clustering

In the unadjusted model, there was no significant statistical association between childhood SES and reporting a single NCD risk factor (Table 4). However, after including control variables in the adjusted model, the odds of reporting a single NCD risk factor were significantly higher among individuals who reported either low (AOR=1.45, 95% CI 1.11 to 2.14) or middle childhood SES (AOR=1.21, 95% CI 1.01 to 2.03) than those with high childhood SES. Similarly, in the adjusted model, the odds of reporting multiple NCD risk factors were observed to be significantly higher among individuals who reported low (AOR=1.88, 95% CI 1.21 to 2.78) and middle (AOR=1.22, 95% CI 1.02 to 2.05) childhood SES compared with those who reported high childhood SES.

**Table 4.** ORs for the association between childhood SES and multiple NCD risk factors, Botswana, 2016

	1 NCD risk factor				≥2 NCD risk factors			
Childhood SES	UOR	95% CI	AOR	95% CI	UOR	95% CI	AOR	95% CI
Low	2.49	0.70 to 4.71	1.45***	1.11 to 2.14	3.63	0.87 to 5.02	1.88***	1.21 to 2.78
Middle	2.52	0.73 to 4.73	1.21***	1.01 to 2.03	1.45	0.56 to 3.97	1.22***	1.02 to 2.05
High	1.00		1.00		1.00		1.00	

*** statistically significant at 5%, reference category is ‘0 NCD risk factors’; UOR, unadjusted ORs; AOR, estimated adjusted ORs controlling for age, gender, education, residence, work status and current wealth status.

### Association between childhood SES and NCD multimorbidity

Unadjusted results for both single and multiple NCD conditions did not show any significant statistical link with childhood SES (Table 5). Meanwhile, after controlling for current SES, multimorbidity was observed to be significantly associated with childhood SES, with individuals from a low SES background more likely to report both single (AOR=1.17, 95% CI 1.00 to 2.01) and multiple NCD conditions (AOR=1.78, 95% CI 1.11 to 2.68) compared with those with a high childhood SES background.

**Table 5.** ORs giving the association between childhood SES and multimorbidity

	1 NCD condition				≥2 NCD conditions			
Childhood SES	UOR	95% CI	AOR	95% CI	UOR	95% CI	AOR	95% CI
Low	0.65	0.04 to 1.98	1.17***	1.00 to 2.01	0.57	0.02 to 1.91	1.78***	1.11 to 2.68
Middle	0.69	0.06 to 2.01	0.78	0.07 to 2.05	1.04	0.01 to 1.96	1.32	0.29 to 1.79
High	1.00		1.00		1.00		1.00	

*** statistically significant at 5%, the reference category is ‘0 NCD conditions’; UOR, unadjusted ORs; AOR, estimated adjusted ORs controlling for age, gender, education, residence, work status and current wealth status; N = 882.

## Discussion and conclusions

The main aim of this study was to use the life course approach to examine the influence of childhood SES on NCD risk factor clustering and multimorbidity among adults in Botswana. The overall prevalence of multiple NCD risk factors and multimorbidity was 30.1 and 5.3%, respectively. These findings are indicative of the effects of the nutritional, demographic and epidemiological transition that Botswana is currently undergoing. Due to rapid changes in unplanned urbanisation, ageing trends, unhealthy dietary patterns, sedentary lifestyles, tobacco and alcohol use, NCD risk factor clustering and multimorbidity are poised to increase considerably in the coming decades in Botswana, replacing ominous infectious diseases such as HIV/AIDS and TB.

Overall, the prevalence of NCD risk factors and NCDs was found to be high among individuals who had low- and middle-level childhood SES. However, after clustering all the NCD risk factors, it was observed that multiple NCD risk factors and multimorbidity were significantly higher among individuals who reported low childhood SES. These findings corroborate previous findings, which found that NCD risk factors such as alcohol consumption, poor fruit and vegetable consumption and smoking are more common among people from low childhood socioeconomic backgrounds.^28–30^ For instance, Enoch^29^ found that poor socioeconomic conditions were strongly linked with an early start of alcohol consumption and smoking, which continues into adulthood. There are possible explanations for the observed association between poor childhood SES and NCD multimorbidity. Adverse socioeconomic and environmental conditions during childhood, including poverty, sexual and physical violence, emotional distress and neglect, can lead to an increased use of tobacco and alcohol consumption during adulthood.^11–13^ Meanwhile, the observed slight variations in childhood SES and poor diets observed in this study may be due to cultural notions that promote non-vegan diets in Botswana. Poor fruit and vegetable consumption predisposes individuals to NCD risk factors and NCDs.

After adjusting for confounders, we found that the ORs of reporting both single and multiple NCD risk factors were significantly higher among individuals who reported low and middle childhood SES than those with high childhood SES. This finding corroborates other previous studies that have linked childhood SES with both low and middle childhood SES compared with high SES.^31–33^ The cumulative effects of poor early life circumstances observed in this study may predispose individuals to a single NCD risk factor at first, with progression to other NCD risk factors over time. This finding is indicative, in the context of the policy debate, because it emphasises the need to recognise the effect of the accumulation of disadvantages that can occur during childhood, which may ultimately lead to inequality in adult morbidity and mortality.

Controlling for current SES, it was found that multimorbidity was associated with childhood SES, with individuals from poor SES backgrounds more likely to report both single and multiple NCD conditions. Similarly, a recent study in Denmark by Jensen et al.^34^ noted that multimorbidity is more prevalent among people of lower SES (both early and later SES). There is further evidence to suggest that socioeconomic inequalities in health persist as advantages and disadvantages accumulate over the lifespan^13^ and that socioeconomic inequality in health becomes gradually smaller over life.^35^ The initial assertion seems to correlate well with the findings of this study that childhood disadvantages accumulate over a lifetime, predisposing individuals to the possibility of multiple NCDs later in life.

The importance of the first 1000 d of a child's life has been opined to be vital for further growth and development of an individual later in life. The government of Botswana has come up with several programmes aimed at reducing child malnutrition. Such programmes include the Destitute Persons Programme, Vulnerable Group Feeding Programme, Orphan Care Programme, Primary School Feeding Programme and Infant and Young Child Feeding, among others. All these programmes are designed to improve the development of individuals during their early years of life to reduce the burden of NCDs in adulthood. Meanwhile, more targeted efforts are needed to reduce child or infant malnutrition, which is a major risk factor that can predispose individuals to NCDs during adulthood.

Although this study provides vital insights on the influence of childhood SES on NCD risk factor clustering and multimorbidity, it has some limitations. First, only association and not causality can be inferred from this study because we used cross-sectional data. Second, this study uses a pseudo-life course approach and does not cover the entire life course of participants. The findings of this study suggest the need for further research on the influence of life course factors on the health of individuals. Consequently, there is a need for longitudinal studies to enhance this understanding.

## Data Availability

Study materials and de-identified data can be obtained from the corresponding author (keetilem@ub.ac.bw) upon reasonable request.
